# Methylcobalamin Facilitates Collateral Sprouting of Donor Axons and Innervation of Recipient Muscle in End-to-Side Neurorrhaphy in Rats

**DOI:** 10.1371/journal.pone.0076302

**Published:** 2013-09-30

**Authors:** Wen-Chieh Liao, Yueh-Jan Wang, Min-Chuan Huang, Guo-Fang Tseng

**Affiliations:** 1 Department of Anatomy, Faculty of Medicine, Chung Shan Medical University and Department of Pediatrics, Chung Shan Medical University Hospital, Taichung City, Taiwan; 2 Department of Anatomy, College of Medicine, Tzu Chi University, Hualien, Taiwan; 3 Institute of Anatomy and Cell Biology, College of Medicine, National Taiwan University, Taipei, Taiwan; "Mario Negri" Institute for Pharmacological Research, Italy

## Abstract

Using ulnar nerve as donor and musculocutaneous nerve as recipient we found earlier that end-to-side neurorrhaphy resulted in weak functional reinnervation after lengthy survival. End-to-side neurorrhaphy however is the sole choice of nerve repair at times and has the advantage of conserving donor nerve function. Here, we investigated whether myelination-enhancing agent methylcobalamin and motoneuron trophic factor pleiotrophin enhances the recovery after end-to-side neurorrhaphy. Methylcobalamin significantly increased the expression of growth associated protein 43 and S100 protein and βIII tubulin in musculocutaneous nerve 1 month after neurorrhaphy suggesting the ingrowth of ulnar axonal sprouts in reactive Schwann cell environment. Upper limb functional test, compound muscle action potential measurements, motor end plate counts, and axon and myelin analyses showed that methylcobalamin treatment alone or with pleiotrophin improved the recovery significantly, 3 and 6 months post-surgery. There were fewer axons, closer in number to that of the intact recipient nerve, found in the distal repaired nerve of the methylcobalamin-treated than that of the vehicle control, suggesting that methylcobalamin facilitates axonal maturation and eliminates supernumerary sprouts. In conclusion, our results showed that methylcobalamin does indeed enhance the recovery of peripheral nerve repaired in end-to-side configuration.

## Introduction

Peripheral nerve injury involving a segmental loss of a particular nerve is a potential indication for nerve grafting and neurorrhaphy. Among the strategies devised over the years, end-to-side neurorrhaphy (ESN) which has the advantage of saving donor functions is a common strategy of the non-maleficence principle for treating nerve lesions. The indication for ESN is for injuries with long nerve gaps and this has been widely explored [1-9]. ESN however induces slow collateral sprouting and requires long postoperative survival for recovery. In animals, this remains unsatisfactory as compared to end-to-end neurorrhaphy (EEN) in a six month study [10] and thus remains debatable [10,11]. Hence, strategies to enhance the outcome of ESN are eagerly awaited.

Vitamin B12 (cobalamin) is important to hematopoietic and nervous tissues [12-15]. The methylated analogue methylcobalamin (MeB12) provides a basis for transmethylation that promotes conversion of homocysteine to methionine and has been shown to have a stronger affinity for nervous tissues than other analogues including cyanocobalamin [12]. MeB12 is prescribed to ameliorate various neuropathies [16,17] and ease the progression of amyotrophic lateral sclerosis [18]. Mechanistically, it has been shown to act on downstream mechanisms of nerve growth factor and brain-derived neurotrophic factor to promote neurite outgrowth as well as the regeneration and conduction of nerves [19]. It has also been shown to have a special affinity for nerve tissues to promote myelination and transport of axonal cytoskeleton [20]. In addition to enhancing axonal regeneration, MeB12 also promotes Schwann cell proliferation and migration [21], which is essential in providing a permissive environment for axonal growth [16]. These make MeB12 a good candidate for facilitating the recovery of peripheral nerve repaired with ESN.

Pleiotrophin (PTN) is a heparin-binding growth factor expressed in developing nervous system and is reported to facilitate peripheral nerve regeneration [22,23]. In rats, PTN had been shown to significantly increase the number of axons growing into the recipient nerve in the EEN of musculocutaneous nerve (McN) to ulnar nerve (UN) [24], therefore we hypothesize that the combination of MeB12 and PTN will enhance the sprouting of intact UN following coaptation of the severed McN.

Here we used the ESN paradigm of coaptating the severed McN to UN with an epineurial window [10] to test the effects of MeB12 alone and in combination with PTN. We examined the expressions of several axonal growth-related markers (Schwann cell marker S100 [24,25], axonal growth marker growth-associated protein 43 (Gap43), and neuron specific cytoskeleton marker βIII tubulin) in the nerve proximal and distal to the neurorrhaphy site to investigate the readiness of reinnervation of target muscle a month after surgery. Electrophysiological, morphological and behavioral means were used to assess the outcome of the combination of drugs up to 6 months following coaptation.

## Materials and Methods

A total of 94 young adult male Wistar rats (Charles River strain, Animal Center of the Medical College of National Taiwan University) aged 6–8 weeks (200-300 g) were studied (Table 1).

**Table 1:** Number of animals studied in each experimental group.

Types of examinations	Experimental groups									
	Sham-operated control	ESN-1 month			ESN-3 month			ESN-6 month		
		PBS	MeB12	MeB12+PTN	PBS	MeB12	MeB12+PTN	PBS	MeB12	MeB12+PTN
Functional tests including CMAP recording and sticker removal grooming test										
Morphology studies including axon counting, axonal diameter and myelin thickness measurements, S100 immunostaining and motor end plate labeling	4	6	6	6	6	6	6	6	6	6
Western blotting of axon growth-related markers	9	9	9	9						

### Ethics statement

Animal experiments were approved by the Animal Care and Use Committee of the Tzu-Chi University under guidelines of the National Science Council of Taiwan. All efforts were taken to minimize animal suffering during and post-surgery.

### End-to-side neurorrhaphy (ESN)

Rats (n = 81) were deeply anaesthetized (8 mg ketamine and 1 mg xylazine/100 g body weight) and prepared for microsurgery. An incision was made along the left mid-clavicular line to expose the UN and McN in the left brachial plexus. McN was transected at the margin of the pectoralis major muscle. An epineurial window matching the cross-sectional size of McN was then made on the UN while minimizing the damage done to the axons. The cut end of McN was attached with 10-0 nylon under a surgical microscope. This configuration of nerve repair could be achieved regularly as shown in detail in our previous report [10]. The wound was then closed with 5-0 silk. Animals were monitored 1, 3 and 6 months following surgery (Table 1). Thirteen sham-operated normal animals were used as control (Table 1).

### MeB12 and PTN administration

Two treatment strategies, MeB12 alone and MeB12 with PTN (MeB12+PTN), were investigated. For MeB12 treatment (500 µg/ml; Methylcobal^®^ Eisai, Tokyo, Japan), 250 µg was injected intraperitoneally [24] every morning for 3 months following ESN. This dosage is in accord with clinical prescription in acquired disorders due to aging, gastric surgery or radiculopathy [26-28]. For the MeB12+PTN treatment, 2 µl of PTN (10 µg/ml; Sigma-Aldrich, St Louis, MO) was injected directly into the nerve at the sutured site immediately after neurorrhaphy, followed by daily MeB12 treatment for 3 months.

### Western blotting

The expressions of an assortment of regeneration-related markers in the neurorrhaphied nerve were examined 1 month following surgery (Table 1). Animals were deeply anaesthetized as described above. A segment of UN and McN immediately proximal and distal to the neurorrhaphied site; approximately 1 cm in length, was excised and homogenized with Kaplan buffer (50 mM Tris, pH 7.4, 150 mM NaCl, 10% glycerol, 1% NP40, and protease inhibitor cocktail) and centrifuged. Equal amounts of cell lysates were separated using electrophoresis (12% SDS-polyacrylamide gel) and transferred onto nitrocellulose membranes. After blocking with 5% skim milk for 1 hour, the membrane was incubated separately with PGP 9.5 (1:1000; Ultraclone; Wellow, Isle of Wight, UK), Gap43 (1:1000; Chemicon, Tamecula, CA), S100β (1:1000; S2532, Sigma-Aldrich) and βIII tubulin (1:1000; Sigma-Aldrich) antibodies at 4 °C overnight, followed by incubation with horseradish peroxidase-conjugated goat anti-mouse IgG or horseradish peroxidase-conjugated goat anti-rabbit IgG (1:10000; Bethyl, Montgomery, USA) as required. ECL reagents (Millipore, Bellerica, MA) were then used to detect the signals generated.

### Sticker removal grooming test

The agility of the affected forelimb was tested using a sticker removal grooming test [24] by placing a small piece of sticker, 1-cm in diameter, to the ipsilateral ear [29]. Grooming was then initiated with a spray of water over the face and the time required to remove the tape was recorded. The test was repeated 5 times and an average score for each animal was derived. The test was terminated if the rat failed to remove the sticker in 5 minutes. All behavior studies were performed by the same blinded observer and analyzed in a double-blind arrangement.

### Compound muscle action potential recording

Compound muscle action potentials (CMAPs) of the repaired nerve and target muscle were recorded with Viking Quest electromyogram (VQ EMG, Nicolet Biomedical, Madison, WI). The repaired nerve and biceps brachii muscle were exposed after anaesthetization. A hand-held nerve locator (Vari-stim®, Medtronic, Minneapolis, MN) was used to confirm the innervation of the targeted muscle before CMAP recording. The stimulating electrode was placed above the reconnection site and the recording electrode was placed in the biceps brachii muscle at the mid-humerus level. The recording electrode was kept 1 cm from the stimulating electrode with a piece of 5-0 nylon suture. The rat’s tail was connected to the signal ground. Following that, the nerve was stimulated with 0.2 msec square pulse current in an increasing magnitude (from 5 to 13 mA) at a repetition of 0.2 Hz. In sham-operated rats, electrodes were placed at corresponding locations on McN and biceps brachii muscle. Data was then digitized and analyzed accordingly.

### Fixation and tissue preparation and analysis

At the end of the survival period, rats were anaesthetized and perfused with 4% paraformaldehyde in 0.1 M phosphate buffer (PB), pH 7.4. Biceps brachii muscle and the repaired nerve were then harvested. Serial transverse sections of the nerves at 3 or 8-µm thicknesses and longitudinal sections of the biceps brachii muscle at 40-µm thickness were made with a cryostat microtome (Leica, Wetzlar, Germany). Selected nerve sections were stained with Hoechst 33342 (1 µg/mL, Sigma-Aldrich) and rabbit polyclonal anti-S100 antibody (1:400, DAKO, Carpinteria, CA) or also with mouse anti-CD68 antibody (1:100, AbD Serotec, Düsseldorf, Germany). Incubation with primary antibody was done overnight at 4 °C. These sections were then reacted with FITC-conjugated anti-rabbit IgG (1:200; Vector, Burlingame, CA) for S100 and Cy3-conjugated anti-mouse IgG (1:200; Jackson ImmunoResearch, West Grove, PA) for CD68 respectively, for 1 hour at room temperature. Muscle sections; one in every 5, covering the largest sectional area, were incubated with rabbit polyclonal antibody against neuronal PGP 9.5 (1:1000; Chemicon, Temecula, CA, USA) to identify nerve fiber and Alexa Fluor 488-conjugated-bungarotoxin to identify motor end plate (MEP) (1:1,000; Molecular Probes, Eugene, OR). PGP 9.5 was then tagged with Cy3-conjugated anti-rabbit IgG (1:400; Jackson ImmunoResearch, West Grove, PA).

The z-stacked confocal images of the muscle were captured with a Leica TCS SP5 confocal microscope (Leica Microsystems) to analyze the MEP clusters and the innervation of biceps brachii muscle. Numbers of innervated MEPs, those with PGP 9.5-immunostained axons, in each of the 15 reacted sections of each muscle were counted and the mean of each muscle was then derived.

### Plastic embedding of the repaired nerve and subsequent evaluation

To count the number and measure the sizes of axons, the fixed repaired nerve distal to the neurorrhaphy site was processed as below. They were immersed in 2% osmic acid solution in PB for 1 hour at room temperature before dehydrated in graded alcohol and embedded in Epon 812 resin (EMS, Fort Washington, PA). The plastic-embedded nerve was sectioned with glass knife transversely at 1-µm thickness, stained with toluidine blue and examined with a light microscope. The numbers and diameters of the axons and thicknesses of myelin sheaths were measured with Image-Pro Plus (Media Cybernetics, Silver Spring, MD).

### Statistics

Prism 5.0 (PRISM, GraphPad Software, San Diego, CA, USA) was used to analyze the results and the data presented are in the form of group mean ± SD. The data were first analyzed for normality with Kolmogorov-Smirnov test. Those qualified (p > 0.1) were then analyzed subsequently with one-way ANOVA followed by Bonferroni post hoc test and a significance level of 0.05 was the criterion. Mann-Whitney U test was only used when normality or equal variance test failed. Sample sizes were determined with G*power 3 [30] where α = 0.05 and power (1-β = 0.8) based on statistics applied in previous study [24].

## Results

End-to-side coaptation of the distal stump of the severed McN to intact UN resulted in innervation of biceps brachii muscle by axon collaterals originated from the latter. The recovery of the PBS-treated following ESN was less than ideal as compared to intact McN, even after 6 months of survival (please see below). These are consistent with our previous report on ESN alone with no treatment.

### Effects on CMAP and upper limb functional recovery

**Figure 1:**
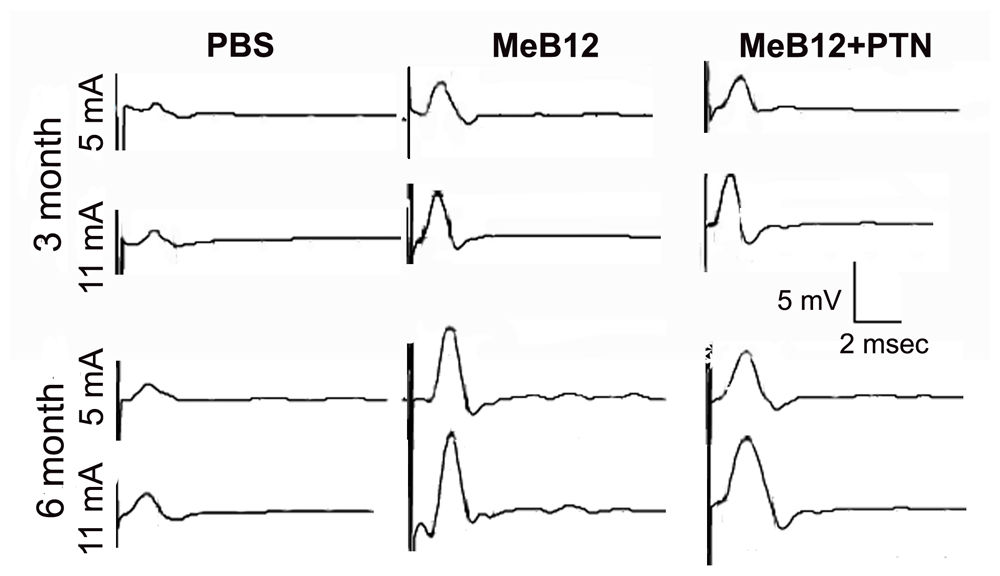
Effects of MeB12 and MeB12+PTN treatments on CMAP. The responses were recorded from biceps brachii muscle upon activation of the nerve. Representative responses recorded from the PBS (vehicle), MeB12 and MeB12+PTN-treated rats 3 (upper row) and 6 months (lower row) following ESN were illustrated. Stimuli at moderate (5 mV) and high (11 mV) strengths were applied to the nerve above the neurorrhaphy site.

We first explored the electrophysiological evidence of the reinnervation of biceps brachii muscle by examining the recipient nerve-evoked CMAP. In normal animals, stimulating the nerve to biceps brachii muscle, namely McN, at 5 mA generated a short-latency CMAP which is comparable in amplitude to that elicited by 11-mA stimulus (Table 2). This suggests that in normal animals most motor units can be readily activated at moderate stimulus strength of 5 mA. However, CMAP could only be marginally identified with higher stimulus strength 1 month following ESN. In the PBS-treated, such evoked CMAPs had long and variable latencies suggesting the development of weak and marginally synchronized motor units. CMAPs could be consistently evoked and had slightly increased amplitudes but remained relatively long in duration in rats 3 and 6 months after surgery (Figure 1, Table 2). This suggests that newly established motor units are weak and poorly synchronized.

**Table 2:** The effects of MeB12 and MeB12+PTN treatments on CMAP of the repaired nerve and biceps brachii muscle 3 and 6 months following ESN.

	Stimulus strength	CMAP amplitude (mV)			
			PBS	MeB12	MeB12+PTN
Sham-operated control	5 mA	5.73 ± 0.20			
	11 mA	6.33 ± 0.47			
ESN-3 month	5 mA		1.20 ± 0.12	3.16± 0.33*	2.85 ± 0.45*
	11 mA		2.40 ± 0.31	4.33 ± 0.63*	4.55 ± 0.7*
ESN-6 month	5 mA		2.20 ± 0.16	4.20 ±1.45*	4.00 ± 1.63*
	11 mA		2.66 ± 0.02	5.00 ± 0.63*	4.80 ±1.28*

CMAPs were recorded from biceps brachii upon stimulation of McN at the stimulus strength indicated. Values are means ± SD. * P < 0.05 between the marked and corresponding PBS-treated (One-way ANOVA followed by Bonferroni post-hoc tests).

CMAPs in MeB12-treated rats a month following surgery had long and variable latencies and generally could be consistently triggered at high stimulus strength (not shown). Similar responses were recorded in the MeB12+PTN-treated rats (not shown). Shorter latency and higher amplitude CMAPs were recorded in the MeB12-treated rats 3 months after surgery. This was also observed in the MeB12+PTN-treated group (Figure 1, Table 2). However, by 6 months post-surgery, the amplitudes of CMAPs recorded in the MeB12 and MeB12+PTN-treated groups with 11 mA stimuli were still slightly lower than the sham-operated control (Table 2). These suggest that the repaired nerve-muscle connection has weaker maximal power than that of the normal muscle. Notice that in the MeB12-treated rats, CMAPs recorded with either 5 or 11 mA stimulus had characteristically short duration (Figure 1, compared the positive phase of the MeB12-6 month traces with others) suggesting that MeB12 enhances the maturation of the reestablished motor units so that they had higher conduction velocity and were better synchronized upon activation.

Behaviorally, MeB12 and MeB12+PTN-treated rats performed significantly better than PBS-treated rats in the sticker removal test as early as 3 months following ESN (Table 3). Like the sham-operated controls, both groups of rats removed the tape in an average of 1 second while the PBS-treated rats took approximately an average of 2.67 seconds to accomplish 6 months following ESN (Table 3). This suggests that MeB12 and MeB12+PTN treatments could enhance the recovery following ESN so that the repaired nerve and muscle were functionally competent.

**Table 3:** Sticker removal grooming test.

		PBS	MeB12	MeB12+PTN
Sham-operated control	1.00 ± 0.50			
ESN-1 month		38.50 ± 12.20	31.20 ± 3.76	35.67 ±10.20
ESN-3 month		35.67 ±10.15	7.00 ± 2.10*	1.00 ± 0.05*
ESN-6 month		2.67 ± 1.86	1.00 ± 0.0	1.00 ± 0.52

Time, in seconds, required to remove a sticker placed on ipsilateral ear was recorded. Values are means ± SD. * P < 0.05 as compared to corresponding PBS-treated (One-way ANOVA followed by Bonferroni post hoc test).

### Effects on the expressions of axonal growth-related markers during early stage of reinnervation

**Figure 2:**
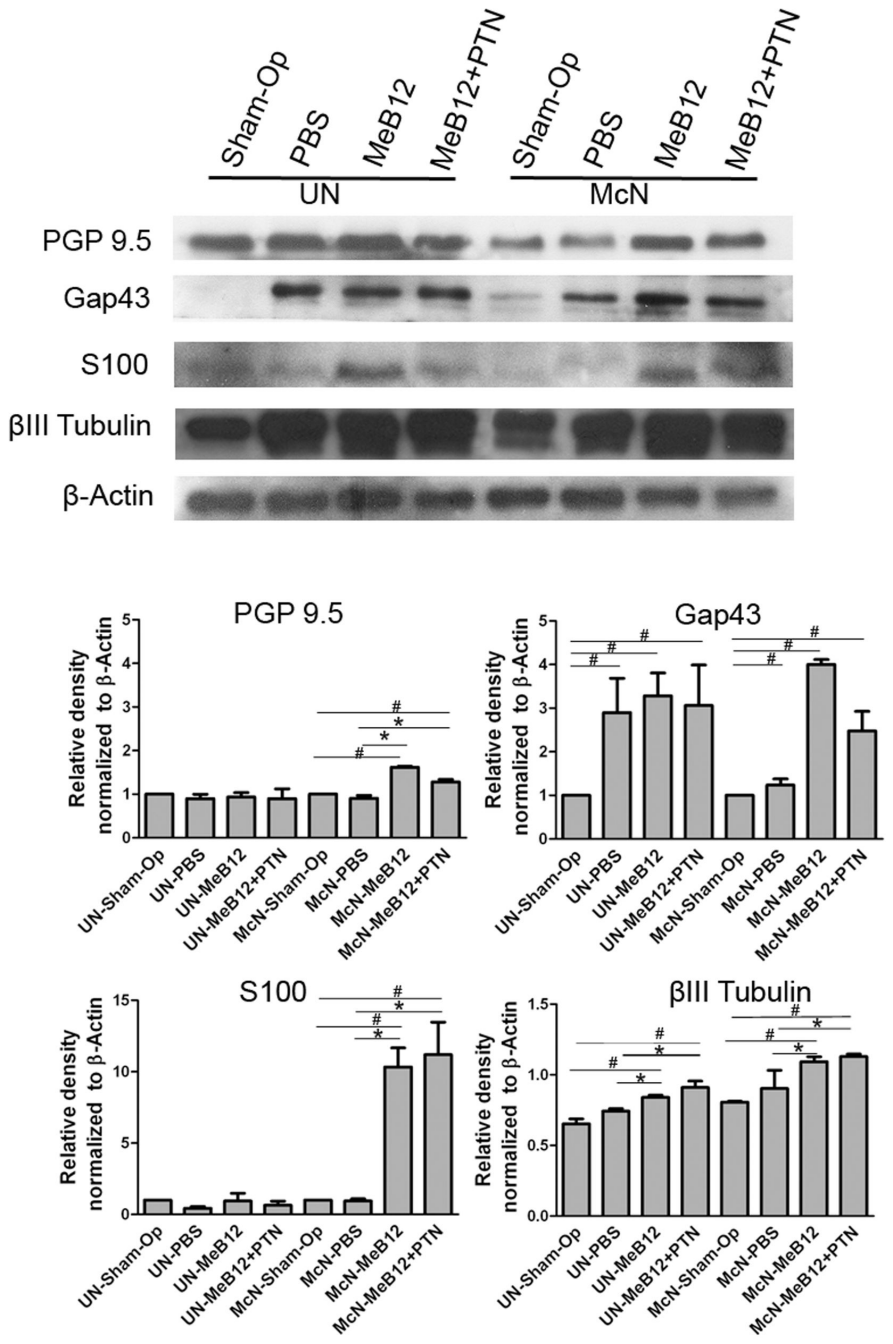
Western analysis of PGP 9.5, Gap43, S100 and βIII tubulin expressions in the repaired nerve 1 month following ESN. The donor (UN) and recipient (McN) parts of the repaired nerve, i.e., the segment proximal and distal to the neurorrhaphy site respectively, were studied. Data from sham-operated (Sham-Op) and PBS, MeB12 and MeB12+PTN-treated rats were analyzed. Expressions of PGP 9.5 (24 kDa), Gap43 (43 kDa) and S100 (11 kDa) were detected. β-Actin is the loading control. Quantifications of the normalized densities of these proteins are shown in the lower half of the figures. Data plotted are mean ± SD (error bar). Statistical significance was determined with one way ANOVA followed by post hoc Bonferroni test. # and *, p < 0.05 between the marked and sham-operated control and ESN-PBS-treated, respectively.

The above functional examinations revealed that differences in the recovery between treatment groups were difficult to discern during early post-surgery period. To resolve this, we looked into the expressions of 3 nerve regeneration-related markers in the repaired nerve 1 month following ESN (Figure 2). The repaired nerve was divided into donor and recipient parts, corresponding to the segment proximal and distal to the neurorrhaphy site. On the donor side, Gap43, an axon-regeneration marker, had increased to around 2 folds above that of the sham-operated control in all ESN animals disregarding whether they were MeB12, MeB12+PTN or vehicle-treated. This suggests that coaptation alone induced the collateral sprouting of donor axons. On the recipient side, Gap43 expression in McN was slightly increased in the PBS-treated but close to 4 folds in the MeB12-treated rats and 3 folds in the MeB12+PTN-treated rats (Figure 2). These are consistent with the proposition that MeB12 effectively facilitates the growth of axonal sprouts into the recipient nerve. Enhanced growth of axonal sprouts into the recipient nerve was also evidenced by the significantly increased expressions of neuron-specific cytoskeleton molecule βIII tubulin in the MeB12 and MeB12+PTN-treated groups and PGP 9.5 in the MeB12-treated group over that of the PBS-treated ones (Figure 2). Expressions of the Schwann cell marker S100 in the recipient nerve of the MeB12 and MeB12+PTN-treated groups was also dramatically increased, around 12-13 folds over that of the PBS-treated (Figure 2). This is consistent with a role of Schwann cells in assisting peripheral nerve growth and myelination.

### Effects on Schwann cells

**Figure 3:**
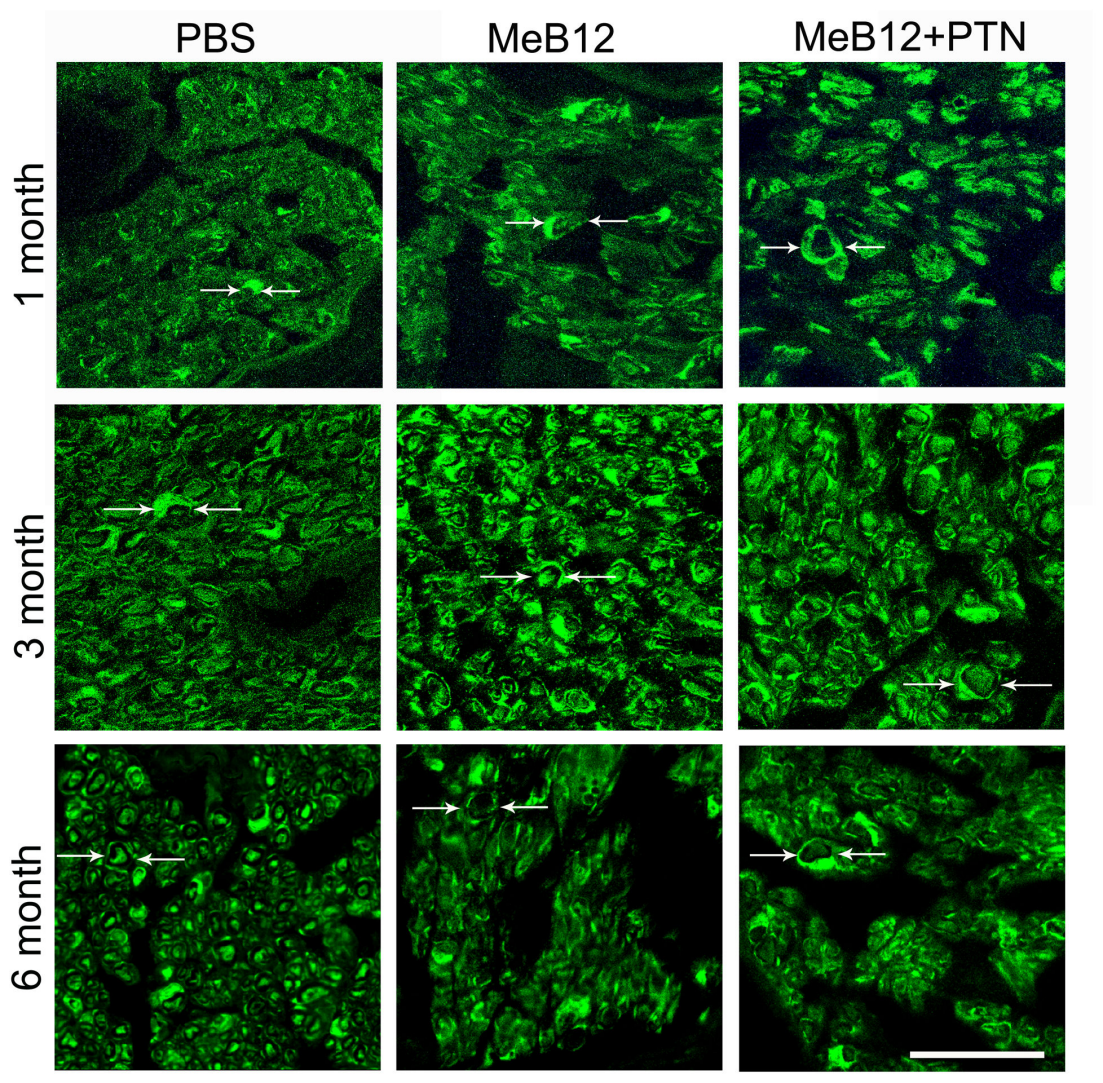
S100 immunoreactivity in the recipient part of the ESN repaired nerve. Micrographs from rats treated with PBS, MeB12 and MeB12+PTN 1, 3 and 6 months post-surgery are illustrated. S100 immunoreactivities (FITC fluorescence) were located in presumably Schwann cells which were seen to encircle axons (between arrowheads). Each confocal image illustrated is the stack of a series of scans in a nerve cross section. Scale bar = 50 µm for all.

**Figure 4:**
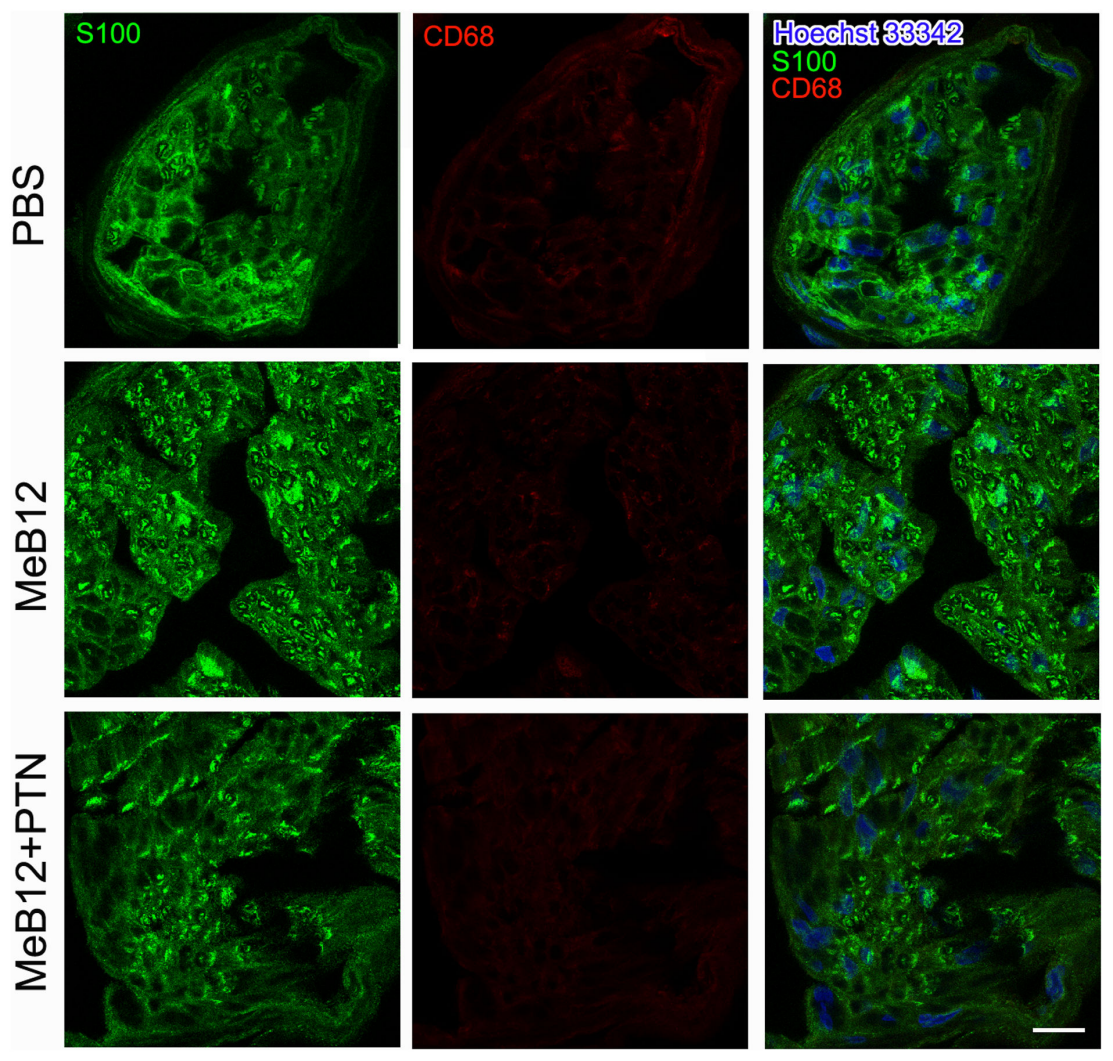
S100, CD68 and Hoechst 33342 labelings in the recipient nerve 1 month following ESN. Micrographs from the PBS, MeB12 and MeB12+PTN treated rats are illustrated. S100 immunoreactivities (green) were found to surround Hoechst 33342-labeld nuclei (blue), presumably Schwann cells. There were little or no detectable CD68 labeling (red) in the recipient nerve demonstrating that most cells in the recipient nerve at this stage were involved in nerve regeneration than degeneration. Each confocal image illustrated is the stack of a series of scans of the nerve section, 8-µm thickness, examined. Scale bar = 50 µm for all.

We also looked into the expression of S100 in cross sections of the recipient nerve using immunostaining method. S100 immunolabeling often appears circular, resembling the Schwann cell surrounding axon (arrows, Figure 3). As expected, brighter S100 labeling were seen in the recipient nerves of the MeB12 and MeB12+PTN-treated group than the PBS control 1 month following ESN (Figure 3). Triple labeling of sections of the recipient nerves 1 month following ESN showed little or no labeling of the macrophage marker CD68 in an abundance of S100 labeling surrounding the Hoechst-labeled nuclei (Figure 4). These suggest that most axonal degeneration-associated debris-removing activities were already over 1 month following ESN. S100 labelings were more intense in the MeB12 and MeB12+PTN-treated groups 3 and 6 months following surgery (Figure 3, comparing the middle and right column panels to those of the PBS-treated column in the left).

### Effects on the numbers, sizes and myelination of regenerated axons

**Figure 5:**
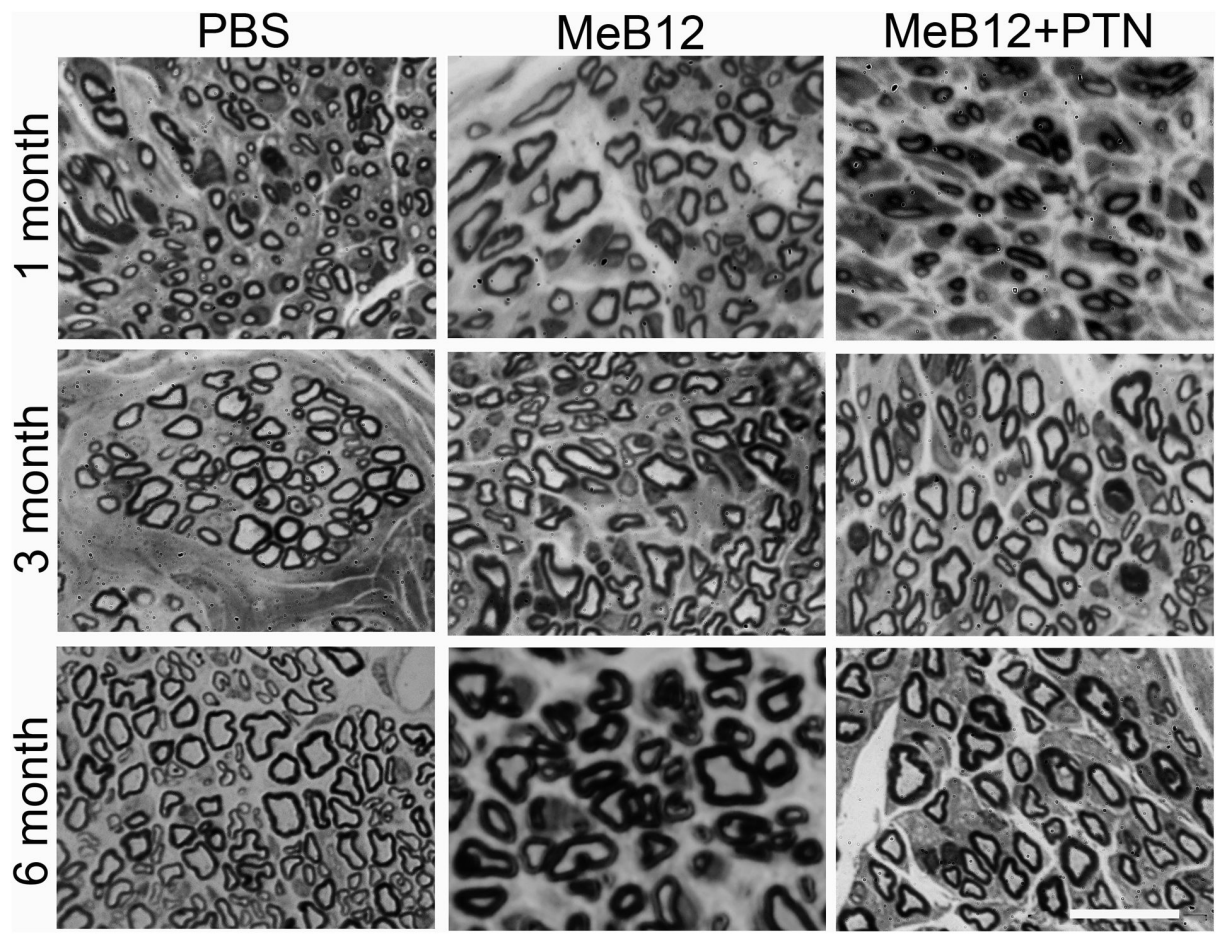
Cross sections of the distal segments of the ESN repaired nerves. Representative micrographs of animals 1 (upper row), 3 (middle row) and 6 months (lower row) following ESN were illustrated. Each micrograph was taken from a toluidine blue-stained, semithin cross section of the recipient nerve 1 mm distal to the neurorrhaphy site. The nerve was embedded in plastic. Micrographs from PBS, MeB12 and MeB12+PTN-treated ESN animals are illustrated. Scale bar = 25 µm for all.

In order to find the anatomic correlates of the effects of treatments we first counted the number of axons entering each recipient nerve. Coaptation alone resulted in the significant growth of the axonal sprouts into the recipient nerve (Figure 5, Table 4) within a month. Each ESN-repaired recipient nerve contained one large-sized vessel a month after surgery disregarding whether it was treated or not (not shown). In the PBS-treated ESN rats, the number of axons was actually far more numerous than that found in intact McNs of the sham-operated animals (Table 4). This is consistent with our finding that the donor, namely ulnar nerve contains more axons than the recipient nerve and this further suggests that trimming of excess axons is required later in the reinnervation process. In the recipient nerve of the PBS-treated group, the number of axons continued to increase 3 months after ESN. Although it did decreased by a small amount, it remained high by the end of 6 months. Interestingly, MeB12-treated rats had fewer axons (21% less) than that of the PBS-treated ones 1 month after ESN (Table 4). The number of axons did increase moderately by the end of 3 months but was 25% less than that of the PBS-treated ones (Table 4). This value decreased by the end of 6 months and was 37% less than that of the PBS-treated rats (Table 4). These phenomena suggest that MeB12 promotes axonal maturation and pruning of redundant axons. However in the MeB12+PTN-treated group, more axons were found to enter the recipient nerve 1 month after ESN compared to MeB12 or PBS-treated group. This value however, decreased by the end of the third month and remained steady 6 months after ESN (Table 4). The early surge of axons in the MeB12+PTN-treated suggests that the PTN applied transiently promotes axonal sprouting.

**Table 4:** Number of nerve fibers in the McN distal to the neurorrhaphy site.

		PBS	MeB12	MeB12+PTN
Sham-operated control	626 ± 224			
ESN-1 month		834 ± 75	656 ± 14*	934 ± 57
ESN-3 month		1186 ± 64	887 ± 28*	723 ± 125*
ESN-6 month		1022 ± 37	643 ± 13*	720 ± 197

Number of myelinated nerve fibers in cross sections of the nerve segment distal to the repaired site was counted. The nerve was plastic-embedded, prepared into semithin sections and stained with toluidine blue. Control nerve was taken from sham-operated animals at corresponding locality. Values are means ± SD. * P < 0.05 as compared to the PBS-treated of the same survival respectively (One-way ANOVA followed by Bonferroni post hoc test).

**Figure 6:**
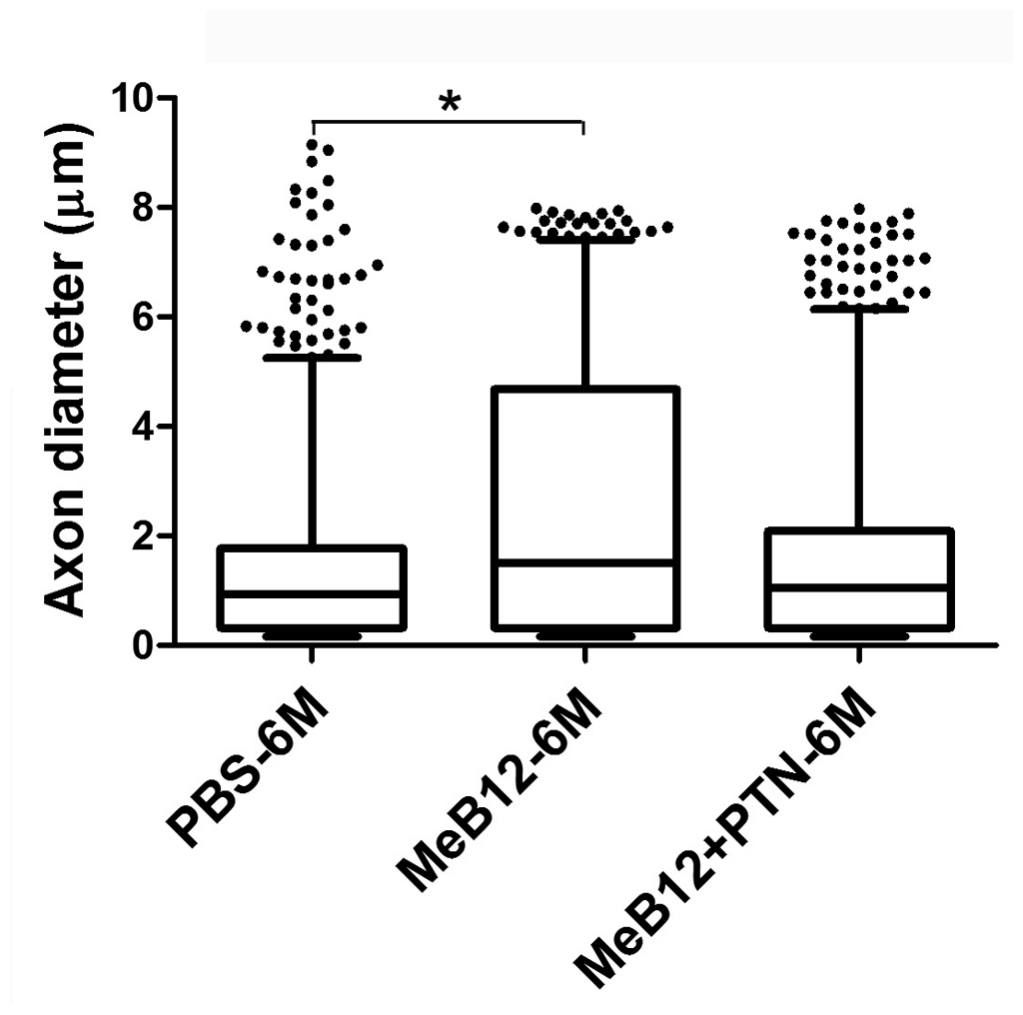
Whisker box plot showing differences in the distribution of the diameters of axons in the recipient nerves of the PBS, MeB12 and MeB12+PTN-treated groups 6 months after ESN. Boxes show 25-75% data ranges. Horizontal lines within boxes show median values. Whiskers show 5-95% ranges and dots show data points outside the 5-95% ranges. Group comparisons were performed with Bonferroni’s multiple comparison tests. *, P < 0.05.

We then quantitated the diameters and myelin thicknesses of the axons growing into the recipient nerve. In the PBS-treated, axons were fine in diameters in the first month and had increased in diameters and myelin thicknesses steadily with survival (Table 5). The mean value of the axon diameters showed that axons of the MeB12-treated were more than twice the thickness than those of the PBS-treated 1 month post-surgery and gained in diameter steadily afterward. In addition, they were always larger than those of the PBS-treated counterparts at any given time point. Like the MeB12-treated group, MeB12+PTN-treated rats also showed a similar-pattern of augmented axonal size growth over that of the PBS-treated rats but the sizes of axons were slightly smaller at each stage. Figure 6 showed a Whisker box plot of axons according to their diameters 6 months following ESN. It shows that the MeB12-treated group had significantly larger diameters than the PBS-treated ones (P < 0.05). The upper outliners of the MeB12-treated group clustered at around 8 µm whereas those of the PBS-treated group varied widely.

**Table 5:** Diameters and myelin thicknesses of nerve fibers in the distal stump of the neurorrhaphied nerve.

		PBS	MeB12	MeB12+PTN
ESN-1 month	axon diameter	1.32 ± 0.02	2.90 ± 0.08*	2.34 ± 0.08
	myelin thickness	0.40 ± 0.01	0.48 ± 0.02	0.46 ± 0.01
ESN-3 month	axon diameter	2.81 ± 0.02	3.50 ± 0.15	3.56 ± 0.33
	myelin thickness	0.49 ± 0.02	0.56 ± 0.04*	0.62 ± 0.02*
ESN-6 month	axon diameter	4.00 ± 0.01	5.20 ± 0.22	4.50 ± 0.94
	myelin thickness	0.78 ± 0.02	1.20 ± 0.06*	1.24 ± 0.04*

The diameters and myelin thicknesses of axons were counted from toluidine blue-stained, plastic-embedded, semithin cross sections of the neurorrhaphied nerve. Values are means ± SD. * P < 0.05 as compared to corresponding PBS-treated (One-way ANOVA followed by Bonferroni post hoc test).

**Figure 7:**
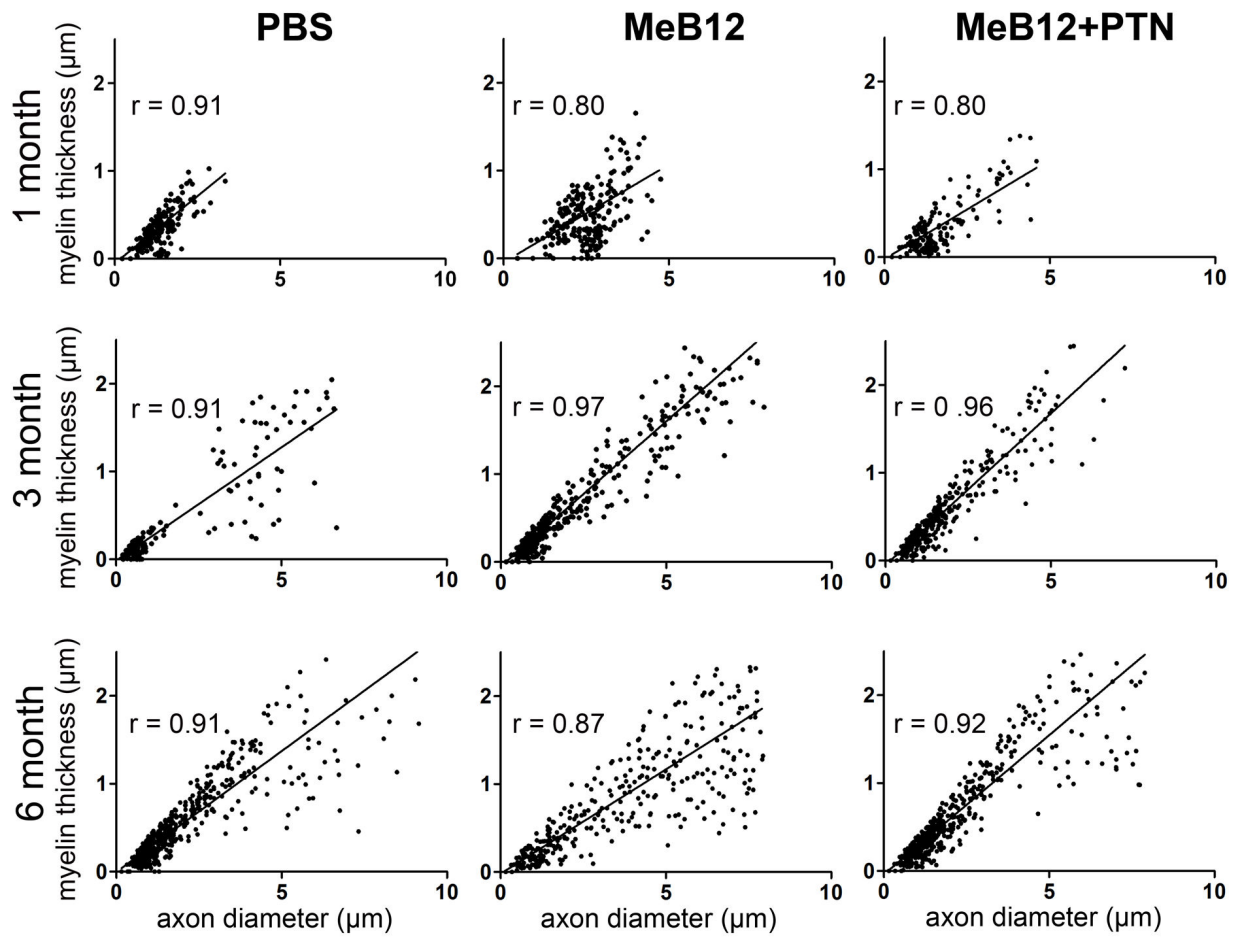
The relationship between axon diameters and myelin thicknesses in the ESN repaired nerves. Representative plots of rats 1 (upper row), 3 (middle row) and 6 months (lower row) following ESN are illustrated. Diameters and myelin thicknesses of all axons in representative toluidine blue-stained sections such as those illustrated in Figure 5 were measured and plotted. A linear regression line that best fits the data points was included in each plot. R represents the residual of the regression illustrated.

On the other hand, mean myelin thicknesses of the MeB12 and MEB12+PTN-treated groups were found comparable to each other but significantly thicker than those of the PBS-treated group 3 and 6 months after ESN (Table 5). These were consistent with the proposition that MeB12 facilitates axonal maturation. Figure 7 plotted the correlation between diameters and myelin thicknesses of the axons growing into the recipient nerves. PBS-treated had predominantly smaller-sized axons throughout the survival period examined. MeB12 treatment increased the sizes and myelin thicknesses of the axons proportionally beginning in a month and the distribution pattern became dramatically different from that of the PBS-treated group 3 months following surgery. Similar pattern of correlation was observed in the MeB12+PTN-treated group but the MeB12-treated group had more points scattered in the upper right sector of the plot, i.e., more larger axons with thicker myelins (Figure 7, the 3-month and 6-month plots).

### Effects on MEPs

**Figure 8:**
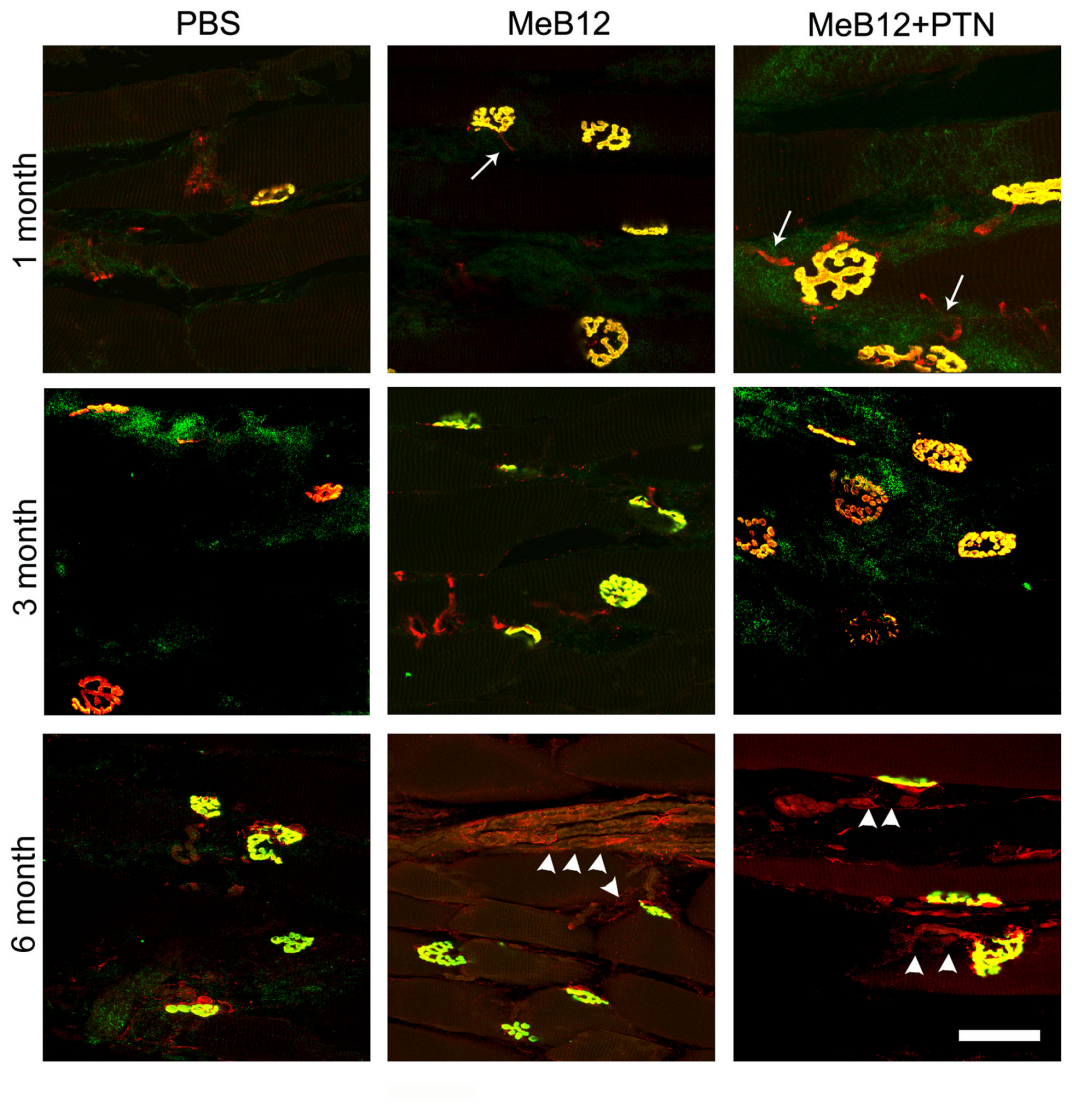
MEPs and innervation of the biceps brachii muscles in rats 1, 3 and 6 months following ESN. MEPs were revealed with α-bungarotoxin tagged with Alexa Fluor 488 (green). Nerve fibers, namely intramuscular axons, were revealed with PGP 9.5 immunohistochemistry (Cy3 red fluorescence). MEP cluster appeared as flower-like structure. Most of them overlapped with nerve staining to become yellow or orange in color. Relatively thin red fibers (arrows) were observed occasionally in the MeB12 and MeB12+PTN-treated muscles 1 month after surgery. Thicker red-staining structures, likely bundles of thicker axons (arrowheads), were seen in the muscles of the MeB12 and MeB12+PTN-treated rats 6 months post-surgery. The fine grain green background staining is the noise from connective tissue covering muscle fibers. Each micrograph illustrated is the stacked confocal scanned image of a portion of a representative muscle section. Scale bar = 30 µm for all.

In order to investigate how muscle innervation recovered over time, biceps brachii muscle sections were reacted simultaneously with α-bungarotoxin tagged with fluorochrome for MEP (green, Figure 8) and PGP 9.5 immunohistochemistry for axons (red, Figure 8). Flower-like MEP clusters (Figure 8) were identified starting 1 month following ESN. Colocalization of nerve fibers in MEPs made most of them appear yellow to orange in color. In the MeB12 and MeB12+PTN-treated, some MEP clusters were often seen to connect to relatively thin red-staining axon-like profiles (arrows, Figure 8) as early as 1 month after coaptation. Large bundles of apparently thicker axons (arrowheads, Figure 8) were found traveling in the muscles of the MeB12 and MeB12+PTN-treated groups 6 months after ESN. To quantitate the efficacy of reinnervation, number of MEP clusters in longitudinal section of each muscle was counted. The results showed that, 1 month following ESN, number of MEP clusters in the MeB12-treated had restored to approximately 72% of that of the normal control as compared to 33% in the PBS-treated group (Table 6). Numbers of clusters grew steadily with survival and reached a level equivalent to that of the sham-operated control in the MeB12 and MeB12+PTN-treated groups 6 months following nerve repair (Table 6). Those in the vehicle-treated however, remained much fewer (Table 6).

**Table 6:** Numbers of MEP clusters per muscle section.

		PBS	MeB12	MeB12+PTN
Sham-operated control	12.00 ± 1.41			
ESN-1 month		3.93 ± 0.56	8.66 ± 0.57*	6.66 ± 0.57*
ESN-3 month		5.14 ± 2.44	10.00 ± 3.46	9.33 ± 3.51
ESN-6 month		6.70 ± 0.96	12.67 ± 1.57*	12.00 ± 2.67*

Numbers of MEP clusters were counted from longitudinal sections of the biceps brachii muscle stained with α-bungarotoxin tagged with fluorochrome to reveal MEP and PGP 9.5 antiserum for axons. Values are means ± SD. * P < 0.05 as compared to sham-operated control (One-way ANOVA followed by Bonferroni post hoc test).

## Discussion

EEN and ESN are used to repair injured peripheral nerves. The two strategies are empirically different in which EEN involves the regeneration of transected donor nerve whereas ESN deals with the collateral sprouting of the intact [10,31,32]. ESN could be in part induced by humoral factors released by the recipient nerve as collateral sprouts of the donor were found capable of crossing a gap between the donor and recipient nerves which were brought close to each other with a Y-shaped silicone tube without suturing [31]. Thus, it’s not surprising to find that ESN results in slower recovery and weaker connection strength [10]. However it has the advantage of saving donor function. In the present study, we found that MeB12 effectively facilitated the outcome of ESN in the form of establishing reasonable strength connection in a shorter period of time as compared to the vehicle-treated.

### Effects of MeB12

In the rat ESN paradigm that we investigated, donor UN motoneurons have been confirmed with retrograde tracer to sprout into the recipient nerve and innervate the target muscle ([10]: figures 1 and 7). The results in the present study showed that coaptation of a severed nerve to an intact one alone is sufficient to induce the latter to sprout. This is supported by the increase of Gap43 expression in the donor nerve 1 month after surgery disregarding treatments, and consistent with earlier report that Schwann cells alone could induce the collateral sprouting of intact axons [31]. Although axonal counts show that the PBS-treated contained more axons in the proximal part of the recipient nerve, more sprouted axon collaterals appear to have ventured distally in the MeB12 and MeB12+PTN-treated groups as only the recipient nerves of these latter two groups showed large increases of Gap43 and βIII tubulin expressions. This is also supported by an increase of PGP 9.5 expression, which is preferentially associated with neuronal cytoskeleton [33] in the MeB12-treated over that of the PBS and MeB12+PTN-treated 1 month after coaptation. These findings are consistent with earlier report that MeB12 promotes the transport of axonal cytoskeleton [20]. The large increase of S100, by and large a Schwann cell marker [10,24], in the recipient nerves of the MeB12-treated group also echoed the enhancement on axonal growth as Schwann cells can induce collateral sprouting of intact axons [31] and are critical to peripheral nerve growth after ESN [25]. Furthermore, it is consistent with earlier reports that MeB12 enhances Schwann cell proliferation and migration [21,34] as well as maturation [24]. The scarcity of the expression of the macrophage marker CD68 in the recipient nerves of both the drug and vehicle-treated groups as early as 1 month after coaptation supports that these Schwann cells are more likely involved in axonal regeneration than degeneration. Thus MeB12 could have dual effects on both axons and Schwann cells that work together to facilitate the reinnervation after ESN. The communication between regenerating growth cones and Schwann cells via the release of acetylcholine from the former and the expression of corresponding receptors in the latter in the process of regeneration [35] could play an important role in establishing an effective innervation in the MeB12-treated group that we explored.

MeB12 treatment enhanced the final outcome of ESN but not the excessive enumeration of invading collaterals. The recipient nerves of the MeB12-treated group contained as many axons as the intact recipient nerve 1 month after coaptation, which is much fewer than those of the PBS-treated. Analyses of axonal numbers, sizes and diameter-myelin thickness relationship suggested that MeB12 boosts the maturation of ingrowing axons to establish effective connection so that larger axons prevailed as the rats’ survival lengthened. This maturation effect is likely to involve elimination and/or pruning of axons which developed weak to no neuromuscular connection. This is consistent with our earlier findings that the distal part of the recipient nerve contained fewer axons, 30-40% less, than its proximal part consistently from 2 to 6 months following ESN without treatment [10]. Elimination and/or pruning of axons are also supported by the early transient increase and later reduction in axonal numbers in the MeB12 and MeB12+PTN-treated groups in the present study. This effect of MeB12 is likely critical to enhance the outcome of neurorrhaphy as hyperinnervation and/or polyinnervation are detrimental to functional recovery [36]. The remaining axons, although less numerous than those of the vehicle-treated, developed effective and robust connections to generate sizable CMAPs upon stimulation and to move the affected upper limb in removing the sticker in the modified grooming test. MeB12 could have altered methylation-related kinase activities and/or oxidative stress-reactive cascades in both neurons and Schwann cells [19,20] to result in such a reinnervation enhancing effect. The precise mechanism however remains to be elucidated.

### Effects of PTN

In the EEN of UN and McN, PTN alone or in combination with MeB12 was found to increase the number of blood vessels in the recipient nerve to two folds of that of the PBS controls which contain 3-4 vessels per nerve [10]. In EEN, MeB12 alone slightly increased the number of vessels while enhancing axonal sizes but not the quantity. These suggest that MeB12 lacks a dominant angiogenic effect. In the present study, one central vessel was identified in all nerves repaired with ESN 1 month post-surgery regardless of the treatments. Hence, it is arguable that MeB12 enhanced the recovery by promoting angiogenesis. Our results instead support the notion that the number of blood vessels in the nerve is determined by the need of blood supply by the amount of tissue. The lack of difference in the number of blood vessels in the ESN repaired nerve treated additionally with PTN supports that a single dose of PTN applied to the nerve right after surgery, followed by continuous MeB12 supply did not enhance angiogenesis although PTN was thought to have such an effect [37].

In EEN of McN to UN in rats, PTN alone was found to enhance the sprouting of the axotomized donor axons for at least 3 months [24]. However at the same time, PTN interacts with heparan sulfates or chondroitin sulfate proteoglycans to inhibit fibroblast growth factor-2 incorporation to Schwann cells [22,38]. This hampers myelination and axonal maturation. Persistent sprouting is also known to impede axonal maturation as neutralizing collateral sprouting improved facial nerve reinnervation [39]. In the present study, PTN+MeB12 treatment increased the sprouting of intact donor axons transiently 1 month following ESN. Decreases in axon numbers afterward suggest domination of axonal pruning and/or elimination subsequently, likely an effect of the continuously administered MeB12. The quick onset of pruning and elimination of redundant and/or weakly connected axons is likely responsible for the development of a sound reinnervation in the MeB12/PTN-treated group. In our earlier studies on the EEN of peripheral nerves, combined PTN and MeB12 treatment was found to postpone axonal maturation and was not recommended for repairing peripheral nerves [10].

### Technical remarks

In this study, several measures were used to assess the recovery following ESN. Numbers of MEP clusters in the affected muscle did not linearly reflect the strength of the re-established innervation as measured by CMAP or predicted by the sticker removal grooming test. This is similar to what we reported earlier when evaluating the outcome following EEN of the same nerves [10]. Thus, counting MEP clusters is not recommended as a sole measure for assessing reinnervation. On the other hand, by the end of the 6th month, rats of the MeB12 and MeB12+PTN groups moved their upper limbs effectively to quickly remove the tape comparable to the sham-operated intact controls. Nevertheless, CMAPs generated by the reinnervated muscle upon high strength stimuli were still somewhat less robust than those of the sham-operated controls. These suggest that CMAP is likely a measure of the maximal power of the newly developed neuromuscular connection.

## Conclusions

We demonstrated in rats that systemic MeB12 effectively enhanced the recovery of UN to McN transfer in ESN configuration. The restoration although somewhat short of that of the sham-operated control for the survival we tested, it appears to be rather effective when compared to the vehicle-treated control. Since MeB12 is used in the clinic to treat peripheral neuropathy, it could be readily adapted to treat ESN. The presumed motoneuron trophic factor PTN [23] however is not recommended as we found no advantage in using it with MeB12. In human, nerve repair is complicated by factors such as delay in treatment, tissue inflammation, and long distance for regeneration. Combinational use of anti-inflammatory drug and MeB12 might be considered as we have suggested earlier for enhancing the recovery of EEN [24]. The effect of combining anti-inflammatory drug with MeB12 in ESN repair however remains to be explored.
